# The Book World of Medicine and Science

**Published:** 1893-07-22

**Authors:** 


### The Book World of Medicine and Science.

### BOOK REVIEWS.

Hypnotism, Mesmerism, and The New Witchcraft. By Ernest Hart. Illustrated, pp. 182. (London: Smith, Elder, and Co.)

Mr. Hart s recent contributions on the later developments of hypnotism have already excited very considerable interest, and we are glad that he has been induced to re-publish his articles in collected form, resulting in this handy and most readable volume. The distinguished author is himself so well versed in all the scientific] phenomena of this hypnotic state, as well as with the unfortunate errors into which its votaries have been betrayed in the past, that his exposure of the frauds which have so recently been presented to the scientific world in the name of science, and;stamped with the prestige of one of the French schools of medicine, is so completely conclusive that so far his trenchant criticisms remain practically unchallenged by the most ardent and enthusiastic hypnotists. But Mr. Hart has succeeded in these few pages in giving a very | lucid and intelligible account of what hypnotism is, as well as what it is not, and his story is told in the racy and conversational style that makes Mr. Hart's discourses such pleasant as well as instructive reading. The time has arrived when everyone who is interested in the current topics of the day must know something of the nature and claims of hypnotism, and we advise everyone who has not already become acquainted with Mr. Hart's articles to enjoy a few hours and improve their minds by reading this book.

### THE JULY REVIEWS.

Criminals have been attracting a good deal of attention lately in the Reviews, both as regards their minds and bodies. The anthropometric system employed in Paris for the identification of prisoners is explained at some length in the New Review by Mr. Spearman, who is a warm advocate "for its introduction at Scotland Yard. The persons under examination are classified in five groups, according to the length and width of head, the finger, the foot, and the fore-arm. A system of sub-divisions, according to the colour of the eyes, &c., supplements these groups and enables the expert in a few minutes to identify with absolute certainty any person previously measured, and this out of a record numbering many thousands.

In the Westminster Review we have the criminal regarded from a pathological point of view by Mr. Hankin. The writer maintains all thinking men admit that crime is a disease in the same sense that lunacy and alcoholism are diseases, and, moreover, that in some the disease is incurable. The prisoner must not be sent to prison for a fixed time as a punishment, he must be compulsorily treated for a dangerous malady. If he can be cured, no expense must be spared in his treatment. All the costly machinery of a kind of moral hospital must be set in motion for him. If incurable, he is to be treated as the lost dog of the human species, "taken to a judicial Battersea, put in the lethal chamber, and painlessly extinguished." There can be no offence in surmising Mr. St. John E. C. Hankin to be a very young man.

There are some interesting recollections of Sir Richard Owen by the Hon. Lionel Tollemache in the National

**THE BOOK WORLD OF MEDICINE AND SCIENCE.—(*continued*).**

REVIEW. The story of the negro's head crops up again in a slightly altered version; it is not a pleasing one in any dress A characteristic trait is recorded of Owen during a visit to Mr. Gladstone at Hawarden. "The host walked about the grounds with his guest, the latter wearing a smart pair of lavender gloves. Full of his favourite subject, Owen suddenly stooped and picked up an unsightly creature (it was either a toad or an uncleanly frog), and the nauseous creature he laid on the palm of his lavender glove. Mr. Gladstone summed up by observing that seldom if ever had any man of science left on his mind such an impression of genius—'not talent merely, but genius.'"

DR. SPRIGGE enters into a curious speculation in the NEW REVIEW on the poisoning of the future considered with relation to bacteriology and alkaloids for which there is no chemical test. It appears there is a widespread apprehension in certain circles that secret poisoning by means of germ inoculation may become the fashion with the up-to-date criminal. "Many people actually believed that the maliciously inclined will be able to send round to the venal doctor—if not to the grocer—for a packet of cholera germs, or to buy hydrophobia 'cultivations' by the pound." This reads like a revival of the mediæval scare about "untori," and illustrates the remarkable manner in which prevailing superstitions reappear after cycles of years dressed to suit the new generation. Dr. Sprigge points out, of course, that the technical skill and scientific knowledge required to manipulate bacteria to any purpose renders the class of persons capable of such elaborate crime exceedingly small. And it may be added that the criminal ranks are rarely recruited from the body of hard-working scientists, whose lives are too full to leave much room for the hazardous enterprise of inoculating their enemies with "ptomaines,'' or "bacillus anthracis."

Discussing the Treatment of Disease by Suggestion in THE CENTURY, Mr. Allan Hamilton describes a process very similar to that of the old German physician quoted above, but for the dissipation rather than the concentration of the enses. "Bearing in mind the soothing effect of monotonous sounds and of the steady dripping of water, . . . I devised an apparatus by which not only rythmical impressions could be made upon the finger tips, but repeated musical sounds were indefinitely evoked from a finely strung catgut by a revolving wheel. I found that it was much easier to produce the hypnotic sleep when the several senses were acted upon at once than when one alone was appealed to.'' Mr. Hamilton does not believe "that a person whose normal condition is one of moral integrity can be made knowingly to commit crimes," but it seems that this danger is being recognized in America, where hypnotism is far more commonly practised than is happily the case in Europe, and in one at least of the States a Bill has been introduced asking for powers to restrain its abuses and furnish safeguards to the public.

---

REFERRING to our notice in last week's issue of "The Travellers' A B C Medical and Surgical Guide," we are asked by Messrs. Burroughs, Welcome, and Co., to state that the book is intended only for the use of those who are out of reach of medical aid, and not in any way as a substitute for such aid where obtainable. The book is only supplied with cases of medicine especially provided for travellers, missionaries, &c.

